# Transcriptional networks of transient cell states during human prefrontal cortex development

**DOI:** 10.3389/fnmol.2023.1126438

**Published:** 2023-04-17

**Authors:** Aditi Singh, Vijay K. Tiwari

**Affiliations:** ^1^Wellcome-Wolfson Institute for Experimental Medicine, School of Medicine, Dentistry and Biomedical Science, Queens University, Belfast, United Kingdom; ^2^Institute of Molecular Medicine, University of Southern Denmark, Odense C, Denmark; ^3^Danish Institute for Advanced Study (DIAS), Odense M, Denmark; ^4^Department of Clinical Genetics, Odense University Hospital, Odense C, Denmark

**Keywords:** cell-fate, cortical development, lineage, transition states, gene perturbation, trajectory

## Abstract

The human brain is divided into various anatomical regions that control and coordinate unique functions. The prefrontal cortex (PFC) is a large brain region that comprises a range of neuronal and non-neuronal cell types, sharing extensive interconnections with subcortical areas, and plays a critical role in cognition and memory. A timely appearance of distinct cell types through embryonic development is crucial for an anatomically perfect and functional brain. Direct tracing of cell fate development in the human brain is not possible, but single-cell transcriptome sequencing (scRNA-seq) datasets provide the opportunity to dissect cellular heterogeneity and its molecular regulators. Here, using scRNA-seq data of human PFC from fetal stages, we elucidate distinct transient cell states during PFC development and their underlying gene regulatory circuitry. We further identified that distinct intermediate cell states consist of specific gene regulatory modules essential to reach terminal fate using discrete developmental paths. Moreover, using *in silico* gene knock-out and over-expression analysis, we validated crucial gene regulatory components during the lineage specification of oligodendrocyte progenitor cells. Our study illustrates unique intermediate states and specific gene interaction networks that warrant further investigation for their functional contribution to typical brain development and discusses how this knowledge can be harvested for therapeutic intervention in challenging neurodevelopmental disorders.

## 1. Introduction

The human brain consists of billions of cells across diverse anatomical yet functionally interconnected regions (Mu et al., 2019). The cerebral cortex is the largest structure in the human brain and is responsible for perception, cognition, and memory-related functions (Cadwell et al., 2019). In the course of evolution, the human cerebral cortex has expanded markedly by more than three times than other closest higher organisms (Yeo et al., 2011; Eze et al., 2021). Cortical expansion underlies the proliferation and upsurge of cellular heterogeneity in specific cortical layers during distinct periods of gestational development (Kriegstein et al., 2006).

The enormous diversity of brain cell types with precise context comes from a pool of neural stem cells (NSCs; Fan et al., 2018; Yang et al., 2022). This progenitor pool of NSCs is known to have several subtypes that are identified in the developing brain from early to late gestation as the cortex matures (Eze et al., 2021) such that they primarily give rise to neurons in early gestation weeks, but as it transforms later in the second trimester, they majorly generate the glial cell populations of the cortex (Fan et al., 2018; Eze et al., 2021).

Neural stem cells in the cortex undergo state transitions in a highly asynchronous fashion to progress towards a specific lineage. During this process, cells undergo several metastable transient states, and characterizing these states is essential to better understand the key steps of cell fate determination during cortical development and how their disruption may predispose to certain neurodevelopmental disorders (Del-Valle-Anton and Borrell, 2022; Singh et al., 2022). This knowledge can be further harnessed for developing targeted therapy approaches.

The single-cell RNA sequencing (scRNA-seq) approach has enabled the investigation of the dynamics of cellular diversity during brain development at an unprecedented scale (Jeong and Tiwari, 2018; Luecken and Theis, 2019). Studies have begun to use the derived knowledge to advance our understanding of lineage relationships (Mayer et al., 2018; Velmeshev et al., 2021). However, much of what is known about cortical development and its regulatory framework has been examined in non-human model systems. Consequently, we do not yet fully understand the intermediate cell transition states and their regulatory gene networks during human brain development (Fleck et al., 2022). It is possible to use advanced computational approaches to reconstruct the developmental trajectories from single-cell transcriptomics (scRNA-seq) datasets (Fletcher et al., 2018). The construction of the lineage trajectories takes advantage of the fact that developmentally related cells tend to share similar transcriptomic profiles. Consequently, lineage approaches can be used to order cells along differentiation trajectories and to study cell fate decisions (Luecken and Theis, 2019). Recent algorithms that model the dynamics of biological processes use the time-series or even snapshot scRNA-seq data to place the cells in the temporal order of lineage development using their gene expression profiles and also identify the intermediate states which are more plastic in nature and important for fate switches (Maclean, 2022). Hence, using state-of-the-art algorithms of lineage evolution and identifying their high-confidence regulatory genetic drivers is an efficient method for selecting novel candidates contributing toward fate transitions.

In this study, we used publicly available scRNA-seq datasets comprising early to late stages of human PFC development to decipher the distinct transient states during the specification of various cell fates and their underlying gene regulatory circuitry. We further reveal that distinct intermediate cell states can reach the same terminal fate using discrete developmental paths. We identified the differential transcriptomic feature of these cell states and further validated key regulators of these features during oligodendrocyte progenitor cells (OPCs) lineage specification, using *in silico* perturbation analysis. Furthermore, we highlight novel gene interaction networks that warrant further investigation in experimental models for their role in cell fate development, maturation, and overall brain functions.

## 2. Methods

### 2.1. Selection and processing of scRNA-seq datasets

There are several single-cell datasets available for the developing human brain with a greater number of cells but none of them covers a range of embryonic days. We attempted to combine the dataset, but owing to several variabilities related to the origin of the lab, sequencing techniques, and platforms, it was better to use a single dataset, and therefore, we selected the data from Zhong et al. (2018). The data gathered involved a smart-seq-based scRNA-seq dataset with 2,309 cells and an average of 2,654 detected genes per cell. Furthermore, it covers a wide range of developmental stages despite a low number of cells having good gene coverage. Notably, we used another dataset from gestation week (GW) 25 with 15,811 cells for human PFC and validated our findings (Bhaduri et al., 2021), which is from the 10X chromium platform with v3 chemistry.

Single-cell data analysis and pre-processing, including data normalization and dimension reduction, were performed using Seurat version ‘4.3.0’ (Hao et al., 2021) in R. First, count matrices of gene expression were imported to Seurat, and following the assessment of QC metrics of datasets, only cells expressing at least 750 genes with expression in at least three cells were taken for further analysis. Here, cells with only a maximum of 10% mitochondrial genes were included. The UMI counts were then normalized for each cell by the total expression, multiplied by 10,000, and log-transformed. Then, the top 2,000 highly variable features were selected, and data scaling was performed before principal component analysis (PCA) on the first significant 20 PCs based on the elbow of standard deviations of PCs. Finally, cell dimensionality reduction was performed through UMAP and cell clusters were annotated into cell types based on markers (Supplementary Table S1) from the source paper in addition to the current updated literature which mostly aligned with the source dataset.

Seurat data was then converted to the python-based format of cell rank (Lange et al., 2022) using SeuratDisk v ‘0.0.0.9020,’ which is an interface for HDF5-Based Single-Cell File Formats. These h5ad files were imported into Scanpy and used for cell rank as well as PAGA in downstream analysis.

### 2.2. Computation of fate probabilities

Initially, to estimate cell–cell transition possibilities, we used CellRank (Lange et al., 2022) and computed initial and terminal states and the fate probabilities of the cell for reaching the terminal states. We used the CytoTRACE kernel of CellRank to reconstruct the cellular trajectory based on cell similarity analysis (Herring et al., 2022). This kernel bypasses the need to define a root cell for pseudotime calculations and predicted the cellular plasticity states based on transcriptional diversity (Shen et al., 2021). CytoTRACE algorithm further feeds this calculated pseudotime into another kernel to calculate a KNN graph where edges indicate the direction of increasing pseudotime and point into the direction of increasing differentiation state. Furthermore, a transition matrix was constructed based on the pseudotemporal ordering and KNN graph, and it was projected onto a UMAP plot. We used the original force-directed layout to plot cells, colored by cell-type clusters.

### 2.3. PAGA method for fate connectivities

To relate the clusters of cells that might be developmentally related to one another, we quantified the connectivity of cell clusters using the partitioned approximate graph abstraction (PAGA) method (Wolf et al., 2019). It is based on the Scanpy (Wolf et al., 2018) package and constructs a k-nearest neighbor graph of cells where partitions are a group of connected cells at a certain resolution *via* the Leiden method. We used the clustering resolution of 0.4–0.6, and the PAGA graph was acquired by combining a node with each partition and linking each node by weighted edges that characterize a statistical measure of connectivity between the nodes or PAGA partitions (Yu et al., 2021). PAGA discarded false edges with low weights and revealed the denoised topology of the global data at the selected resolution. The nodes that did not connect in the PAGA graph are cells that do not have any significant connections at all. The PAGA nodes were then arranged in a desired path as per the observed significance, and cells were ordered in that path to trace gene expression changes along the trajectories. We used differential expression analysis from Scanpy to calculate the highly significant gene in one node in comparison to the rest of the node or PAGA partitions.

### 2.4. Gene perturbations

We used the iQcell platform (Heydari et al., 2022) v.1.1.0 to investigate the gene regulatory networks. It is a program to understand, simulate, and further analyze workable logical gene regulatory networks from scRNA-seq data, which uses gene expression datasets along with their pseudotime profile to infer gene interactions and their regulatory features. Gene regulatory networks allow the simulation of hypotheses leading developmental programs and also infer the direction of regulation, i.e., positive or negative regulation of gene pairs in a network. In the network, genes were placed in a hierarchy as per their expression densities in pseudotime. iQcell requires a discrete form of input for the mRNA levels as on/off, and we used k-means clustering for the discretization of gene expression levels as default. Before running the simulations, we defined the initial state of the simulation through iQcell, which found the initial cell states, i.e., cell genotype from the discretized expression with early pseudo-time value by averaging the state of each gene over the cells.

## 3. Results

**Figure 1:**
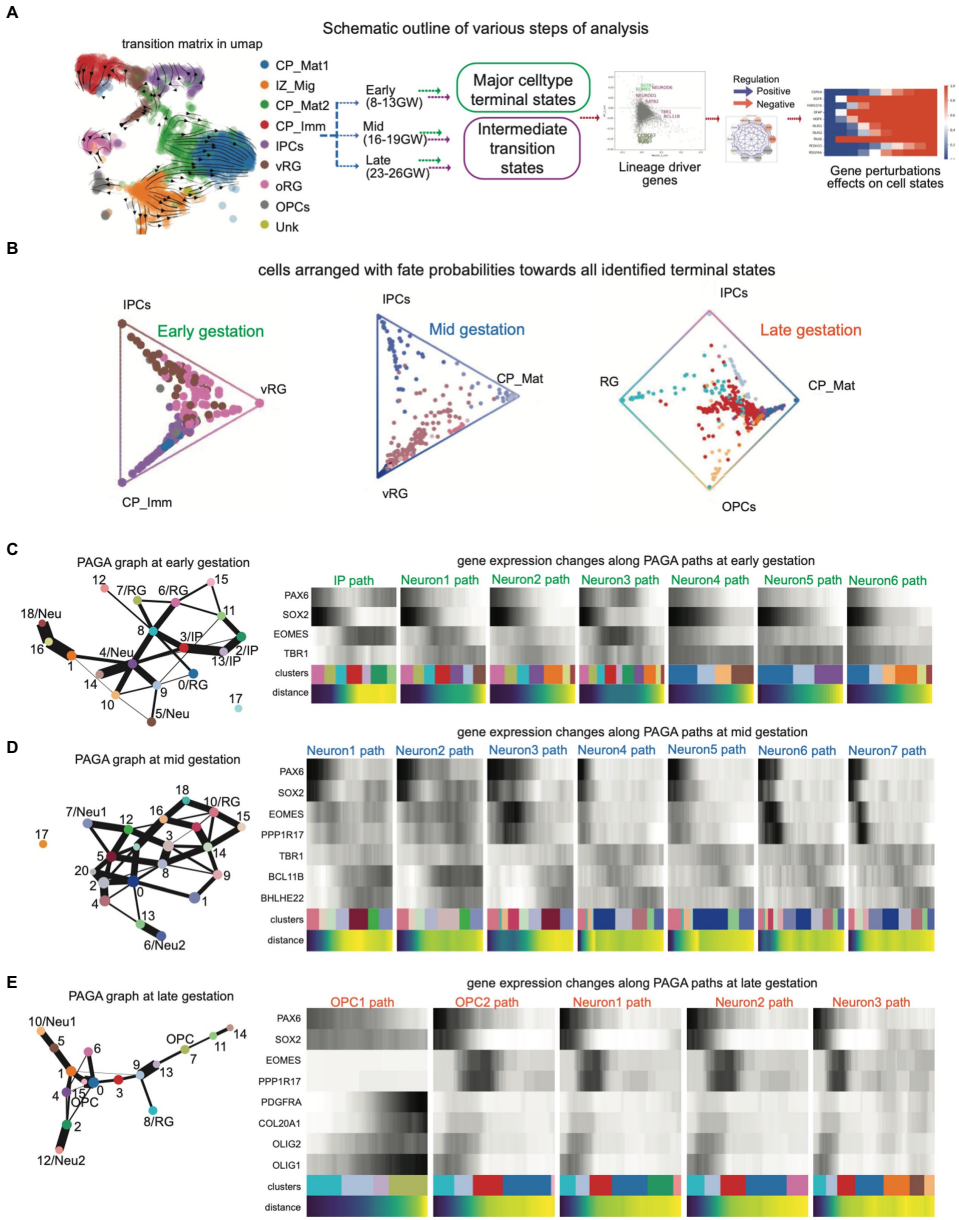
**(A)** Schematic outline of various steps of analysis: The scRNAseq dataset was processed for QC analysis and cluster identification with known cell type markers. Developmental stages were separated into early (8–13 GW), mid- (16–19 GW), and late (23–26 GW) stages and then terminal fates and their intermediate states were analyzed using CellRank and PAGA, resulting in lineage-specific novel genes. Gene regulatory network (GRN) and perturbation analysis were performed using the iQcell platform; **(B)** Summarized fate probabilities toward all terminal states. In this representation, uncommitted progenitors and immature cells can be seen near the center and committed cells are placed near one edge; **(C–E)** PAGA graphs show cell clusters in which only terminal major cell states are named while the rest of the cells are arranged in a graph connected with weighted edges for their nature. The PAGA graphs capture the proximity of progenitors and strong connections within neuronal clusters and then glial clusters; **(C)** PAGA graphs (left) and gene changes along PAGA paths (right) at early embryonic brain development; **(D)** PAGA graphs (left) and gene changes along PAGA paths (right) at mid-stage embryonic brain development; **(E)** PAGA graphs (left) and gene changes along PAGA paths (right) at late-stage embryonic brain development.

We began by using a publicly available dataset that covers gestation week (GW) 8–26 to span key developmental stages with good coverage for distinct cell types (Zhong et al., 2018). The cells were clustered into major cortical cell types using the marker set from the literature (Supplementary Table S1) which matched with the source dataset for broad cell-type classification. The developmental stages were separated into early (8–13 GW), mid (16–19 GW), and late (23–26 GW) stages (Figure 1A). These developmental stages were then revalidated for the proportion of major cell types and then investigated for genetic lineage drivers of the cell state transitions in early, mid, and late stages toward a specific fate.

### 3.1. Distinct lineage transition paths at early, mid, and late gestation times

Biological systems exist in a dynamic state during development with active cell-fate transitions. Computational algorithms can infer the developmental trajectory and predict cell fate by the sequential ordering of cells using their transcriptomic profiles. For such time-based ordering of transcriptomic states in cortical cells, we selected cells that originate within the cortex, while microglia and interneurons, the cell types that have a separate origin and majorly migrate to the cortex, were removed as mentioned previously (Nowakowski et al., 2017; Zhong et al., 2018; Bhaduri et al., 2021).

Then, we used the CellRank method to infer the cell state dynamics in the early, mid, and late stages of gestation. CellRank uses similarity-based trajectory inference with directional information to create probabilistic trajectories in cell fate directions. We identified the probable terminal fates (macrostate) and the calculated probability of each cell to achieve the terminal fates. Subsequently, we created a global map of fate potentials in the form of initial, terminal, and intermediate cell states of the system and assigned each cell the probability of reaching each terminal macrostate (Figure 1B). At early gestation, most of the cells were arranged in vRG (ventricular radial glia) to IP (intermediate progenitor) axis, and few cells could be seen toward immature cortical plate (CP) neurons from IP as well as vRG. Overall, it is clear that most of the cells at early embryonic development are in the center, indicating less committed or plastic states. However, the cells in mid-gestation showed more density on the axis of vRG to mature CP neurons. Interestingly, as we moved to the late gestation stages, we observed the emergence of additional terminal states, i.e., glial population, and interestingly, here the cellular fate probabilities diverged toward the neuronal as well as oligodendrocyte lineage. Moreover, there were many immature cells and/or progenitors from both neuronal and glial lineage which were clustered toward the center.

### 3.2. Fate connectivities provide multiple lineage transition paths toward a terminal cell fate

We then wanted to identify the lineage-specific genes toward neuronal or glial fates and therefore compared the fate probabilities to uncover lineage-specific gene expression patterns and putative lineage drivers. Given that CellRank provided the terminal states and lineage drivers by automated computation of fate probabilities and does not need manual identification of root cell population within CyTOtrace kernel, we questioned if we could find overlapping lineage drivers in transient states through a topology-preserving map of single cells. Therefore, we used partition-based graph abstraction (PAGA) which constructs a simplified representation of the developmental trajectory using a graph-based approach, through similarities and differences between cells based on their gene expression profiles, and then simplifies the graph into a set of clusters or partitions that correspond to distinct cell types. Thus, we achieved the discrete cell types and continuous cell transitions toward these cell types, where both the continuous and disconnected nature of biological cell types are preserved at multiple resolutions (Figures 1C–E). Here, each node represents a cell type and edges measure the strength of connectivity between nodes or the similarity of cell types.

PAGA explores the complex trajectory structure with multiple branching in a general graph form for all embryonic stages (Figures 1C–E), where we identified the root cells and terminal fates (clusters) by specific markers (Supplementary Figures S1–S4). Namely, the root cell was selected using the expression of marker genes for radial glia (PAX6, SOX2; Supplementary Figures S1–S3) and defined the PAGA path as the transitions toward IP (EOMES; Figure 1C; Supplementary Figures S1–S3), different neuronal cells (NEUROD1, BCL11B, and SATB2; Figures 1C,D; Supplementary Figures S1–S3), or OPCs (OLIG1 and PDGFRA; Figure 1E; Supplementary Figure S4). The sequence of transition paths to cell fates is mentioned in Supplementary Table S2. IP and Neuron1 path in early stages, Neuron3 path in mid-stages and OPC1 in late stages show a smooth transition of gene expression profiles. Interestingly, these states contain several transient intermediate cell states, i.e., the PAGA connecting cell clusters which can transition from the root RG cells to the terminal fates, and are of major interest being connected to separate fates with discrete gene expression profiles.

### 3.3. Transition state-specific gene sets at early, mid, and late gestation times

**Figure 2:**
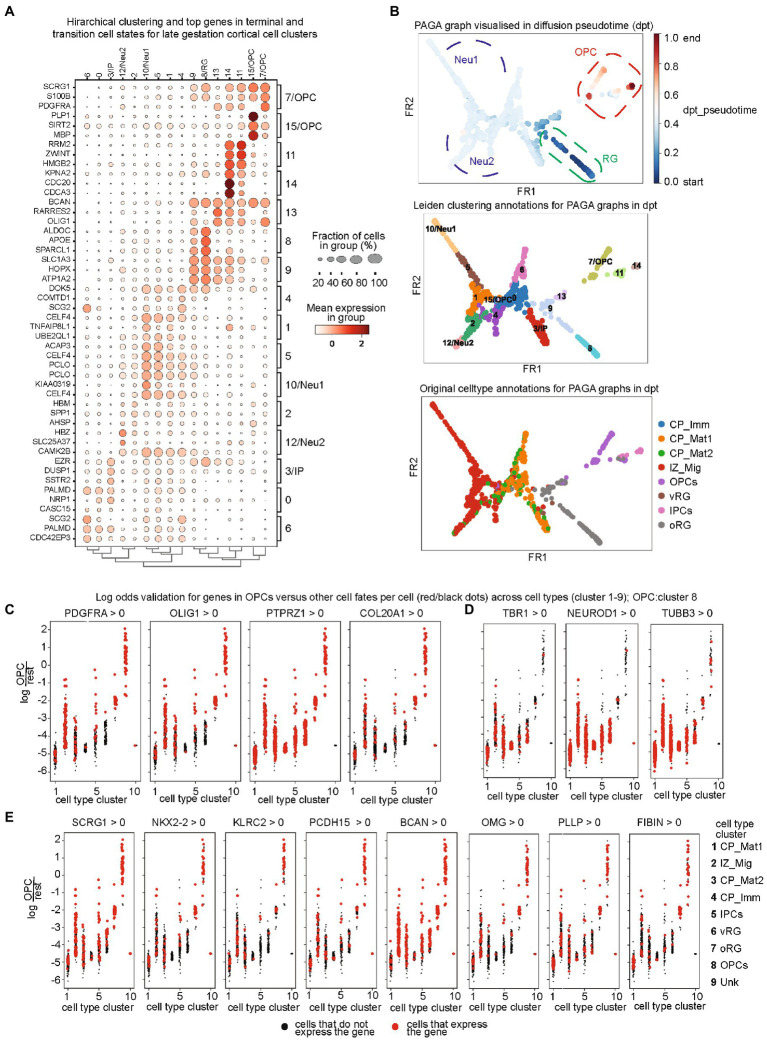
**(A)** Cellular state-specific top gene sets at late gestation time: Hierarchical clustering of identified PAGA clusters and their top genes in terminal and transition cell states for late gestation cortical cells. Fraction of cell numbers is represented in percentage (%), indicated by the circle size and mean expression intensity from low (white color) to high (red); **(B)** Diffusion pseudotime (dpt_pseudotime) plot for PAGA clusters (upper panel), which is then annotated into the PAGA cluster annotations (middle) and then the original cluster annotations from the initial dataset (bottom panel). All annotations show great alignment and confirm the accuracy of diffusion pseudotime. The dpt_pseudotime panel shows the radial glia lineage in dark blue, then neuronal in light blue, and later OPC lineage in red; **(C–E)** Validation of top lineage drivers by plotting the distribution of log odds for OPCs versus other cell fates per cell across cell types. Log-odds ratio confirmed the lineage specificity by comparison of OPC markers genes **(C)**, neuronal genes **(D)**, and the pre-OPC lineage genes.

We next performed a hierarchical clustering of all identified terminal and intermediate cellular states with their variable gene features in the early, mid, and late stages of gestation (Supplementary Figure S5; Figure 2A). As evident in early stages, most cells showed gene expression profile in plastic states in RG and IP states and then transition to the neurons (Supplementary Figure S5A). Furthermore, the later stages showed discrete patterns of cell type (Supplementary Figure S5B; Figure 2C), which is indicative of increased neuronal differentiation. Neurogenesis began early in development and declines during late embryonic development, while gliogenesis was observed in post-mid-embryonic stages and continues in parallel to neuronal maturation and synapse formation till the postnatal stages. We constructed a diffusion pseudotime from PAGA which validates the OPC lineage evolving at a later pseudotime point and hence our choice of root and terminal cell clusters (Figure 2B). Here, we paid special attention to OPCs as there are still critical gaps in understanding lineage drivers of OPC fate switches. We identified top lineage-specific genes for OPC from CellRank and PAGA algorithms and validated top lineage drivers by plotting the distribution of log odds for OPCs versus other cell fates per cell across cell types together on a Seurat clustering global map. Log-odds ratio confirmed the lineage specificity by comparison of OPC markers genes (Figure 2C) and neuronal genes (Figure 2D). The pre-OPC lineage genes (Figure 2E) were also validated by log-odds ratio. In addition, we confirmed their expression in a pseudotime plot for lineage specificity and identified targets of some novel transcription factors using specificity in pseudotime (Supplementary Figure S6). Therefore, these gene are the ideal candidates the ideal candidates for investigating their regulatory effect on OPC lineage commitment and progression.

### 3.4. Perturbation analysis reveals a role for distinct gene interactions in the lineage trajectory of OPCs

We employed the iQcell platform to construct novel gene regulatory networks (GRN) and subsequently investigated the effects of perturbing gene expression states and their effect on cell fates in late embryonic stage scRNAseq data of PFC. In addition, we validated the similar GRNs and perturbation effects in a larger dataset (>15,000 cells) of late embryonic (GW25) PFC.

The first essential step in iQCELL methodology was to calculate gene correlations which later contribute to GRN identifications but scRNA-seq datasets suffer from false negative reads of mRNA or dropout effect that impacts genes with low copy numbers and consequently the gene correlations. Therefore, iQcell uses the Markov Affinity-based Graph Imputation of Cells (MAGIC) algorithm to correct the data for dropout effects and takes advantage of the higher numbers of genes to infer gene network relations (Van Dijk et al., 2018). MAGIC simply computed the affinities between neighbor cells and applied it to recover the undercounted values of individual gene expression.

**Figure 3:**
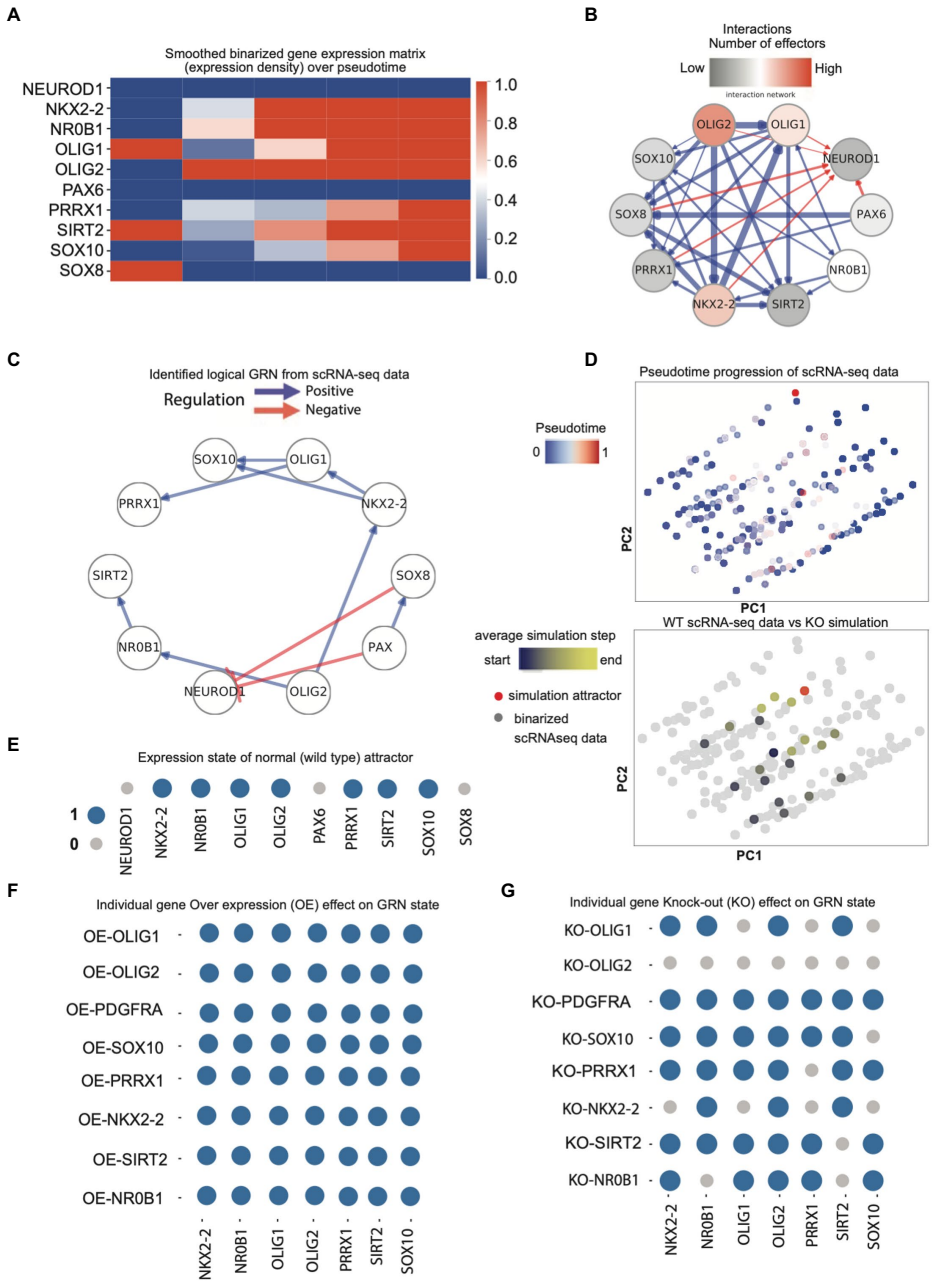
**(A)** Initial smoothed binarized gene expression states for neuronal and OPC genes (expression density). Gene expression values are binarized by clustering, then averaged over a pseudo-time window, and thereafter sorted based on transition points from early to late. Red means high expression and blue means low expression; **(B)** the set of all possible gene–gene interactions, filtered by interaction hierarchy and mutual information. Positive and negative interactions are represented by blue and red edges, respectively. Edge width represents the relative amount of mutual information of the interaction. **(C)** The provisional GRN for OPC lineage genes shows the direction of gene regulation for selected candidates. The GRN is obtained by constraining the possible interactions both to follow the *in vivo* data progression when executed as a logical network and maximize the mutual information between gene pairs. Positive and negative interactions are represented by blue and red edges, respectively. **(D)** Upper panel: The PCA plot of the binarized scRNA-seq data color-coded with the pseudo-time values attributed to each cell. The binarization is performed by clustering the scRNA-seq expressions into expressed or not expressed levels; lower panel: The PCA plot of the simulated developmental trajectories is overlaid on the binarized scRNA-seq. The detected attractor is colored red. The simulated data are color coded by the value of the average simulation step (average distance to the attractor of simulation); **(E)** Expression states of the GRN model steady-state attractor. Genes that are expressed (1) and not expressed (0) are represented with blue and gray circles, respectively; **(F)** Expression states of the model attractors under sequential overexpression (OE) perturbations; **(G)** Expression states of the model attractors under knock-out (KO) perturbations.

After data imputation, we identified the interesting genes for GRN inference in two ways: first, by automated selection plus overlap with PAGA genes and CellRank lineage drivers which mostly aligned with our selection of genes containing OPC lineage genes (Supplementary Figures S6, S7A), and second, by manually curating to keep only transcription factors in top candidates (Figure 3A). In a functional GRN created by iQcell, interactions are not necessarily biophysically direct rather they capture the consequence of regulatory relations.

The iQcell filtered the number of gene interactions through binarization of the gene expression counts clustering into expressed and non-expressed states (Figure 3A; Supplementary Figure S7A) and formed a gene interaction hierarchy. The resulting directional network served as the foundation for inferring executable GRNs (Figure 3B; Supplementary Figure S7B). We then identified the initial cell states of interacting genes for simulating the perturbations (Figure 3C; Supplementary Figure S7C). Initial states were based on interaction networks and regulatory profiles (Figures 3B,C; Supplementary Figures S7B,C), which show ideal OPC profiles for known transcriptional regulators. Moreover, neuronal genes were observed to be blocking the OPC lineage genes and vice versa (Supplementary Figure S7C). Here, the gene interaction networks were assigned the signs (+/−) based on Pearson correlation. Positive means genes are positively regulating each other and negative means one can repress the other. iQcell uses discrete expression levels of genes; therefore, the expression levels of mRNA counts were converted to on/off levels. Although the GRNs here essentially did not show direct modulation, it is interesting to observe how few genes (FIBIN, OLIG2, PMP2, and BCAN) show unidirectional regulation having a single interacting effector gene while other genes in a superior hierarchy such as OLIG1, EGFR, and PCDH15 show more than one effector of the regulation (Supplementary Figure S7C). PAX6 showed autoregulation, which is known to be essential for controlling the balance between neurogenesis and neural stem cell self-renewal (Supplementary Figure S7).

It is further important to consider that these predictive regulatory interactions are based on the temporal gene expression in the lineage, and not all of these predicted genes are transcription factors. Nevertheless, we validated the nature of these interactions in a larger scRNAseq dataset containing 15,811 cells with a total of ~19,000 genes (Supplementary Figure S7D). Here, we could observe some filtering of these interactions and additional negative regulators but there is still retention of interaction patterns between important OPC genes. Then, we focused on our selected TF candidates and their interactions with established OPC lineage regulators in the larger dataset. Expression for these was validated in PAGA-based OPC clusters (Supplementary Figure S8). The resulting TF regulatory networks are quite discrete, where we observed OLIG2 regulating different genes including NKX2-2, which has been implicated in oligodendrocyte differentiation, and it further appears to regulate OLIG1 and SOX10. Furthermore, the negative regulation of NEUROD1 is expected in OPC lineage, but it is interesting to notice the negative regulation of NEUROD1 by SOX8 along with PAX6, where PAX6 appears to be positively regulating SOX8 (Figure 3C). We performed the simulations for normal, gene overexpression (OE) and knockout (KO) conditions and performed the Principal Component Analysis (PCA) to observe any differences (Figure 3D). Based on the role of regulatory genes as observed in GRNs, we simulated the normal OPC profile with the absence of PAX6, SOX8, and NEUROD1 and the presence of the rest of the TFs (Figure 3E, where the blue circle indicates the gene on and gray indicates off and similarly, their state in perturbation type) and performed the sequential perturbations for these TFs to analyze the attractor state that represents the long-term behavior of simulations linked to biological phenotypes.

The principal component analysis of simulated profiles for normal and perturbed conditions shows huge variation in cell states, as well as a developing trajectory in the pseudo time for perturbed KO states (Figure 3D). Gene perturbation effects showed that the overexpression of genes does not largely affect the fate (Figure 3F) or expression state of other genes but knock out does (Figure 3G). The knockout of OLIG2 affected all analyzed gene expression states related to OPC lineage as they are downstream in GRN, while OLIG1 KO caused the absence of SOX10 and PRRX1 as well as NKX2-2, which is upstream to OLIG1. Similarly, NR0B1 KO affected the SIRT2 expression state. We also found that FIBIN, OLIG2, PMP2, BCAN, and SCRG1 have important roles in regulating the lineage but not in deciding the fate as shown in the knockout analysis of all selected genes (Supplementary Figure S7E). These perturbation states provide interesting observations for gene interactions that might affect how OPCs develop into oligodendrocytes and affect their maturation and placement in the brain.

## 4. Discussion

The cerebral cortex is strikingly enlarged in humans and known to be responsible for our mental abilities such as intelligence, cognition, and perception (Reillo et al., 2011; Yeo et al., 2011). Constituent cortical cell types are generated during embryonic brain development through a series of neurogenic and gliogenic processes (Eze et al., 2021). However, the molecular regulatory sequence of the events underlying these developmental processes is less well understood. Therefore, characterizing the cellular and molecular heterogeneity of the human cortex is essential to understand its functional regulations and understand how its disruption may contribute to the emergence of neurodevelopmental disorders.

Cortical cell types express varying transcription factor combinations at distinct phases of development (Singh et al., 2022). These transcription factors (TFs) are critical to specify correct signals and driving the development of distinct neural cell types in the brain. Here, using single-cell transcriptomic datasets of the developing prefrontal cortex from GW8-26 (Zhong et al., 2018) and late gestation stage (GW25) (Bhaduri et al., 2021), we characterized and validated the molecular regulatory features of cell fate transitions at early, mid, and late stages of embryonic brain development. It is known that cell fate decisions are stochastic and more so at the early embryonic development, and at the later time points, more deterministic fates are made as they arise from more committed precursors and a complex regulatory network of TFs, specific to each state. Nonetheless, this transcriptomic complexity almost certainly exceeded several-fold through several transient transcriptomic cell states that exist during development. Understanding cortical development, hence, necessitates characterizing these transient cell states. It has been proposed that these transient cellular states are more plastic in nature and able to undergo specific changes in core gene regulatory programs and enable the specification or conversion into various cell fates (Goldman and Poss, 2020). This is also relevant to illustrate how cortical stem cells can be directed to specific cell fates for disease modeling or treatment. Hence, we calculated the fate probabilities for each cell using the CellRank algorithm on the scRNAseq dataset and ordered the cells in initial, terminal, and intermediate states, taking into account the gradual and stochastic nature of cellular fate decisions. Single-cell transcriptomics (scRNA-seq) has advanced our knowledge concerning cellular heterogeneity and discrete regulatory networks. scRNA-seq captures a snapshot of sequenced cells at one timepoint, but those individual cells can represent a reasonably wide range of dynamic stages or cell states; therefore, it is more practical to arrange them in the temporal order of cell states to infer the trajectory of cell fate transition and identify the intermediate transient states crucial for cell-type specification (MacLean et al., 2018). Consequently, we analyzed the gene expression dynamics with respect to different fates and identified the most prominent regulators of cell fate decisions. Given the terminal states, we computed the probability of how likely a cell will transition toward any of these terminal states and plotted the computed fate probabilities in the force-directed embedding. To understand the cell transition paths in depth, we used the PAGA program and characterized that neural stem cells can reach a cell fate using distinct intermediate states making them traverse a distinct molecular path that might be regulated through a variety of intrinsic and extrinsic signals. By analyzing the gene expression dynamics along each path, it is clearer to see the gradual dynamics of a cell fate and assess the correct or more feasible path with defined genetic circuitry.

The majority of the neuronal cells develop during embryonic brain development, and there is little evidence and still an undergoing debate that neurons are generated in the adult brain. Most of the proliferating cells in the adult brain have oligodendroglial lineage origin that can still proliferate, differentiate, and mature into oligodendrocytes later in the adult brain (Kuhn et al., 2019). In the late stage of embryonic brain development, a spike in gliogenesis and OPC lineage has been detected in abundance during the second trimester of embryonic brain development. Analysis of transcriptional processes has revealed a distinct period of OPC generation in mouse and human embryonic development. Thus, to analyze human cortex-specific regulation of OPC lineage, we explored the regulatory features of certain pre-OPC lineage genes, that were inferred from lineage analysis and already known in OPCs to likely affect the development and maturation (Huang et al., 2020). We used the iQcell platform (Heydari et al., 2022) to infer the gene regulatory network and analyze the effect of gene manipulations on cellular dynamics. These genes were selected as top overlapping features from CellRank (Lange et al., 2022) and PAGA (Wolf et al., 2019) intermediate states as lineage drivers. iQcell modeled the gene interactions as Boolean logic function and then simulated the effect of gene knockout or overexpression as a result of normalizing their level from pseudo time levels. It was evident that only certain factors have a role in lineage commitment while the rest are important in a hierarchical manner for different functions during the differentiation and maturation of OPCs. Overexpression of these genes does not show huge changes and as expected neuronal perturbations do not affect the glial cells and vice versa. Although, initially, the low cell number and especially low cell number per developmental stage tend to affect the analysis outcomes, still the most prominent features are well captured. Furthermore, we ruled out the major concern of low cell numbers and revalidated our observations using a stage-specific higher read and cell number dataset of GW25 with 15,811 cells for OPC lineage GRN identifications and gene perturbation effects.

Along with the characteristic OPC genes, we observed contributions of the novel SOX group TFs toward the OPC lineage, such as SOX8 repressing the neurogenic genes for OPC fate switches and SOX10 being a downstream effector of OLIG1. SOX10 has been widely studied in promoting oligodendrocyte differentiation in humans (García-León et al., 2018) as well as in other species (Takada et al., 2010; Hornig et al., 2013), but the role of SOX8 in OPC cell lineage is not well-defined (Stolt et al., 2005; Wang et al., 2014). OLIG1 and OLIG2 mediated the regulation of certain important TFs such as NR0B1 and PRRX1, and their downstream effectors is an interesting avenue to be explored in OPC maintenance or differentiation to oligodendrocytes. Overall, we established these sequential GRNs in two scRNA-seq datasets and also identified novel TF regulatory networks. We validated that scRNA-seq has the power to identify lineage drivers and that iQcell can filter genes that are not significant in lineage commitment while still having other roles. Therefore, these genes should be investigated for their regulatory roles in future experimental studies.

## Data availability statement

Publicly available datasets were analyzed in this study. This data can be found at https://www.ncbi.nlm.nih.gov/, GSE104276 and https://data.nemoarchive.org/biccn/grant/u01_devhu/kriegstein/transcriptome/scell/10x_v2/human/processed/counts/.

## Author contributions

AS analyzed data and wrote the manuscript. VT supervised the study and wrote the manuscript. All authors contributed to the article and approved the submitted version.

## Funding

This study was supported by the Deutsche Forschungsgemeinschaft TI 799/1-3 and Novo Nordisk Foundation P3110103 grants to VT.

## Conflict of interest

The authors declare that the research was conducted in the absence of any commercial or financial relationships that could be construed as a potential conflict of interest.

## Publisher’s note

All claims expressed in this article are solely those of the authors and do not necessarily represent those of their affiliated organizations, or those of the publisher, the editors and the reviewers. Any product that may be evaluated in this article, or claim that may be made by its manufacturer, is not guaranteed or endorsed by the publisher.
