# Supplementary material for: Transcriptional networks of transient cell states during human prefrontal cortex development
Source: Front Mol Neurosci. 2023 Apr 17;16:1126438. doi: 10.3389/fnmol.2023.1126438 (PMC10150774; doi:10.3389/fnmol.2023.1126438)

Expression of radial glia and intermediate progenitor markers in PAGA clusters at early-gestation age

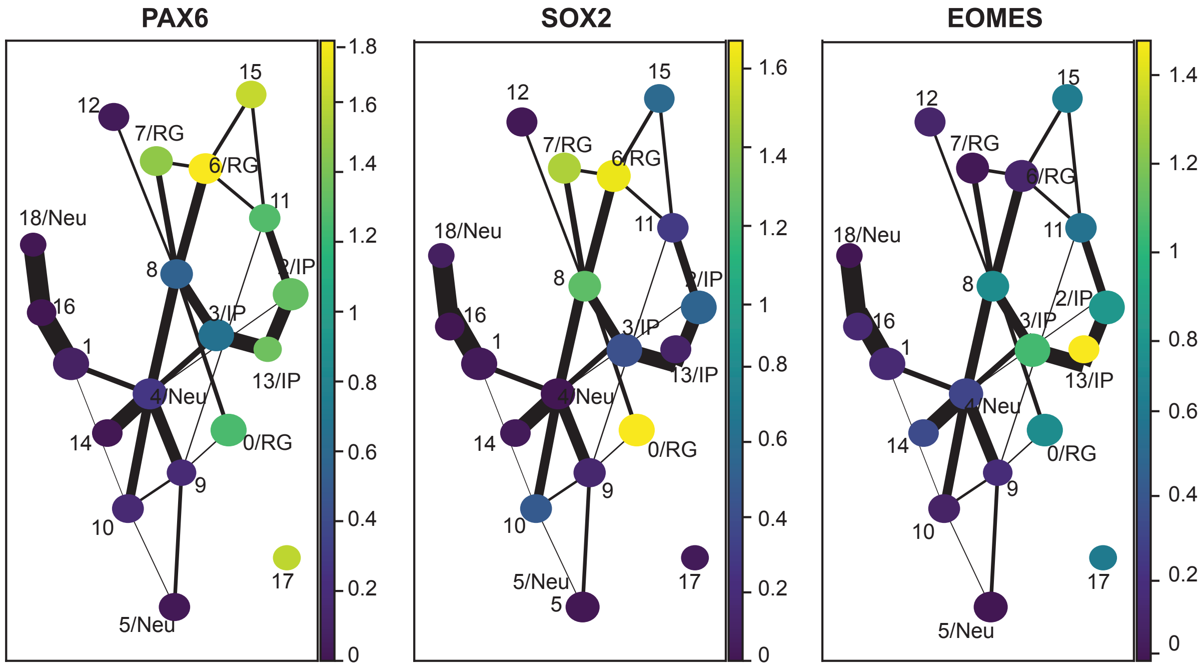

Expression of neuronal markers in PAGA clusters at early-gestation age

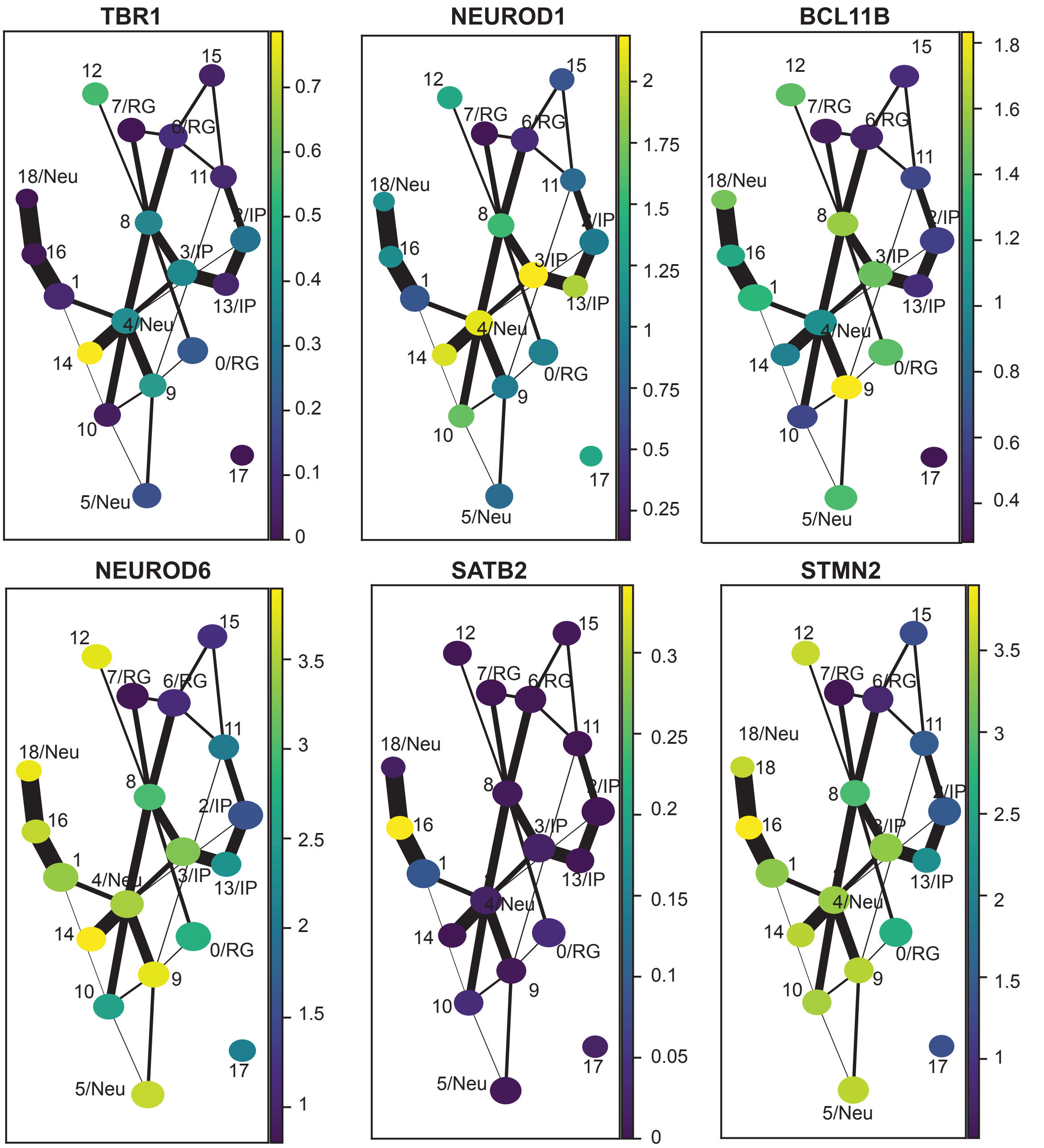

Supplement: Supplementary file 1 [file Image_1.PDF]
