# Supplementary figures and images for: Transcriptional networks of transient cell states during human prefrontal cortex development
Source: Front Mol Neurosci. 2023 Apr 17;16:1126438. doi: 10.3389/fnmol.2023.1126438 (PMC10150774; doi:10.3389/fnmol.2023.1126438)

Fig. S4

Expression of OPC marker genes in PAGA clusters at late-gestation age

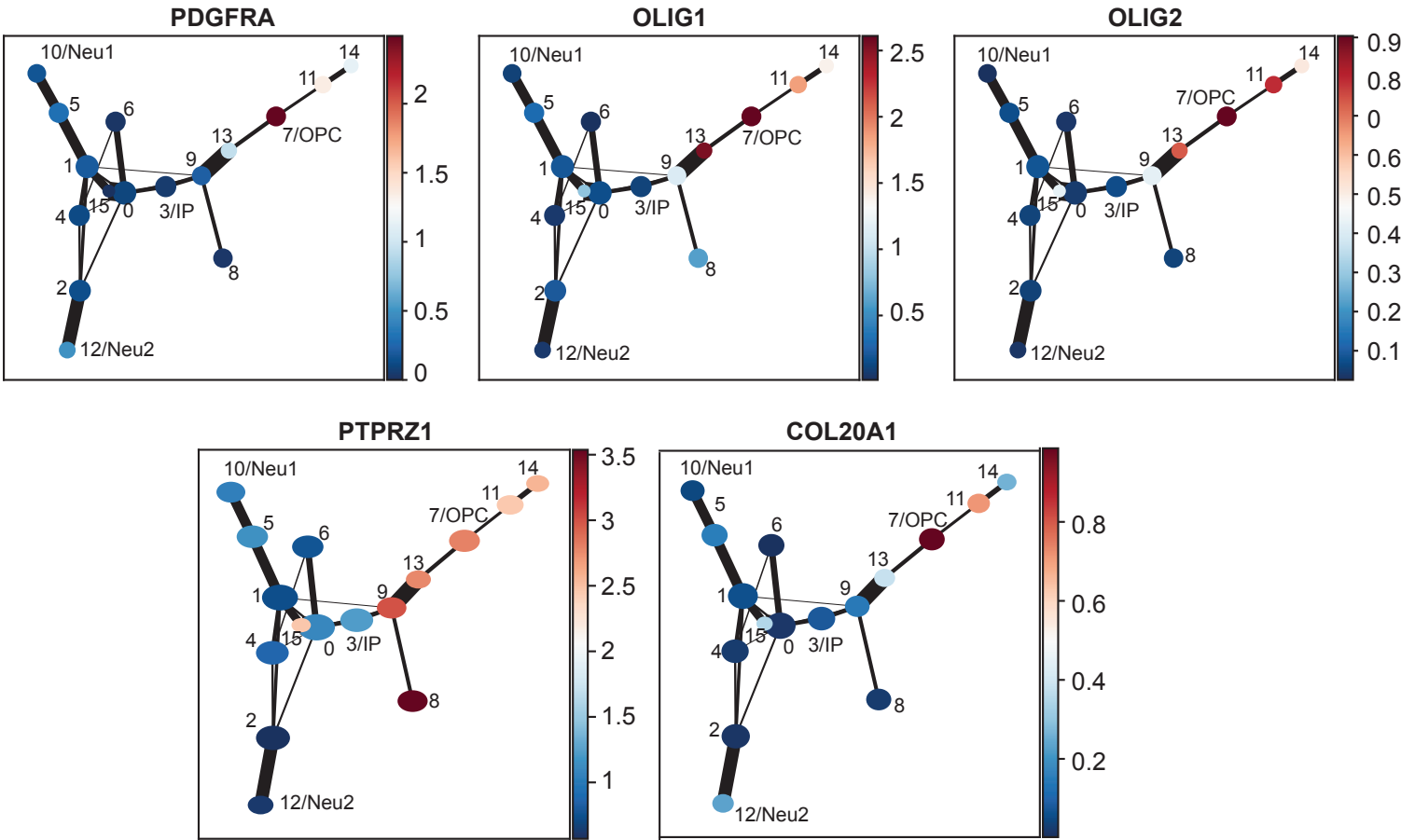

Supplement: Supplementary file 4 [file Image_4.PDF]

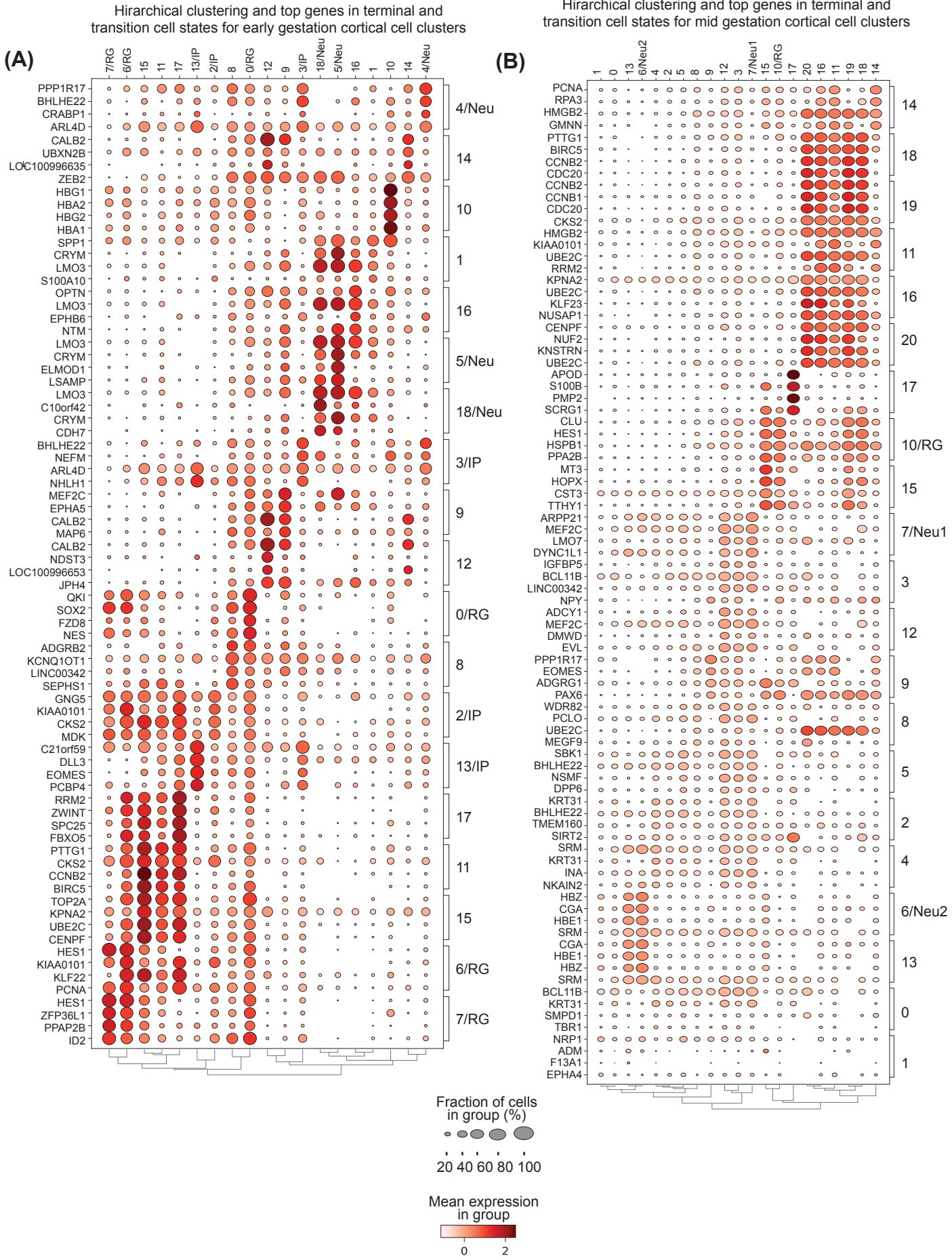

Supplement: Supplementary file 5 [file Image_5.PDF]

Fig. S6

Expression of OPC lineage genes in diffusion pseudotime

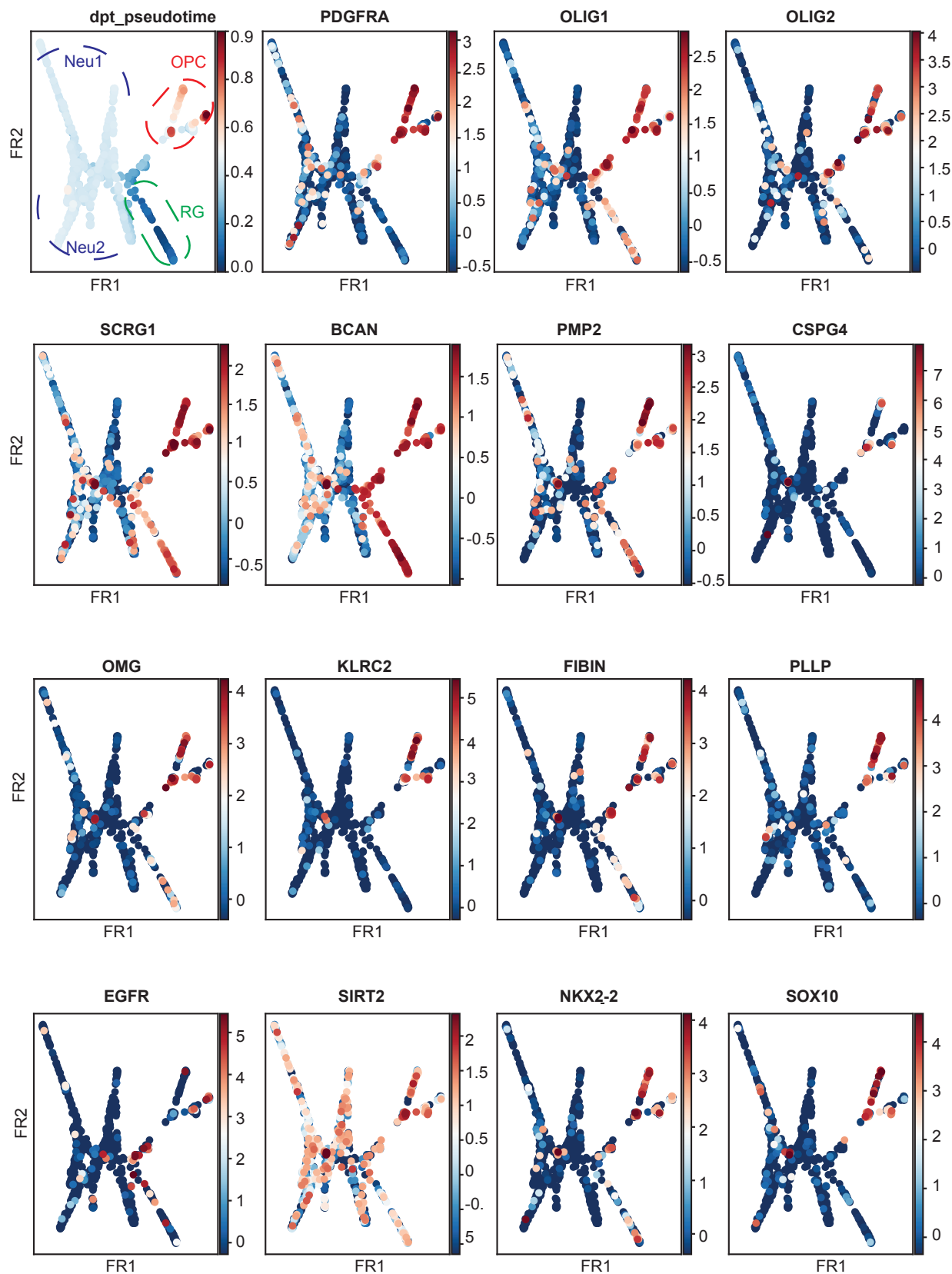

Supplement: Supplementary file 6 [file Image_6.PDF]

Singh et al.

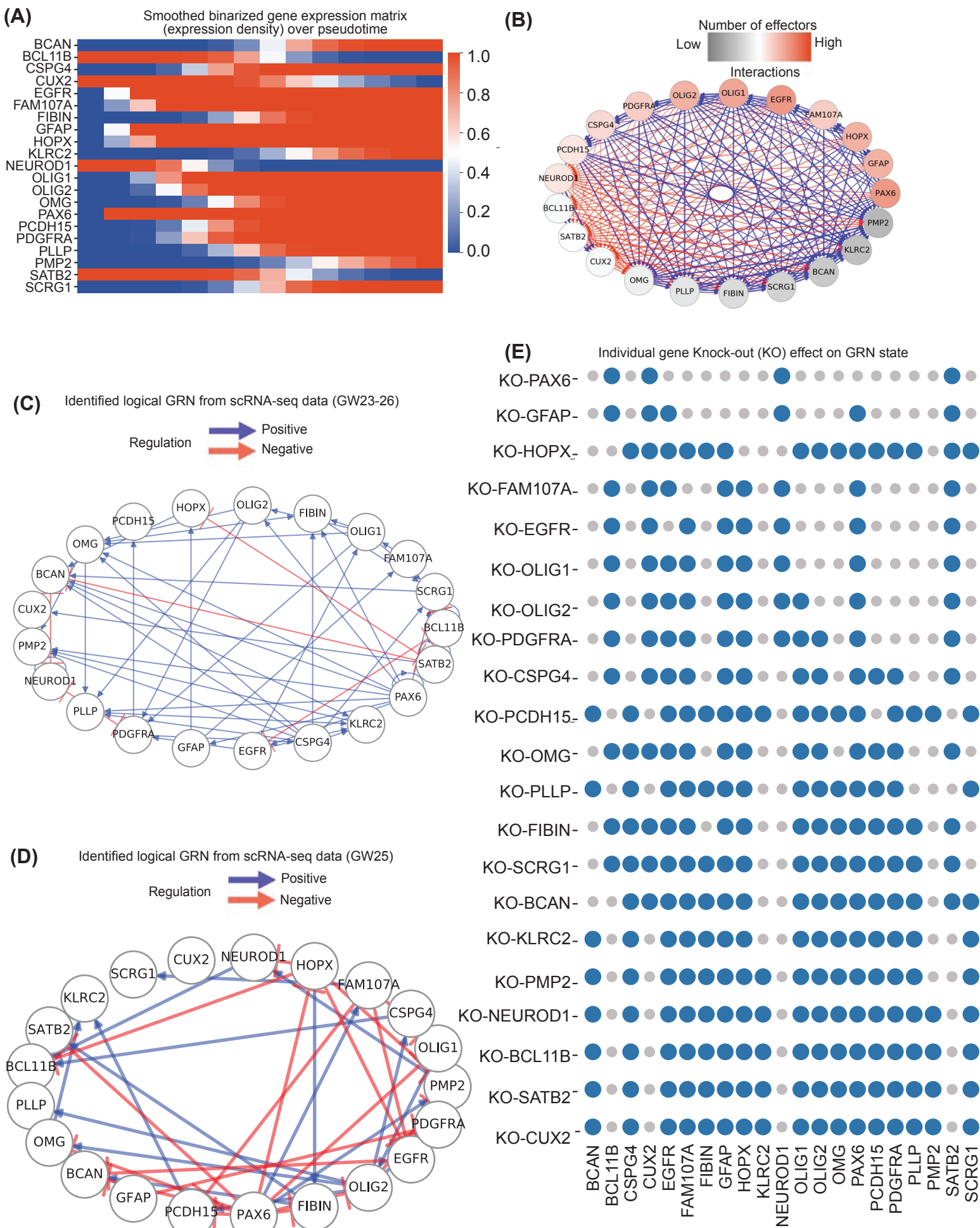

Supplement: Supplementary file 7 [file Image_7.PDF]

Fig. S8

Expression of identified OPC regulatory genes in PAGA clusters at late-gestation age

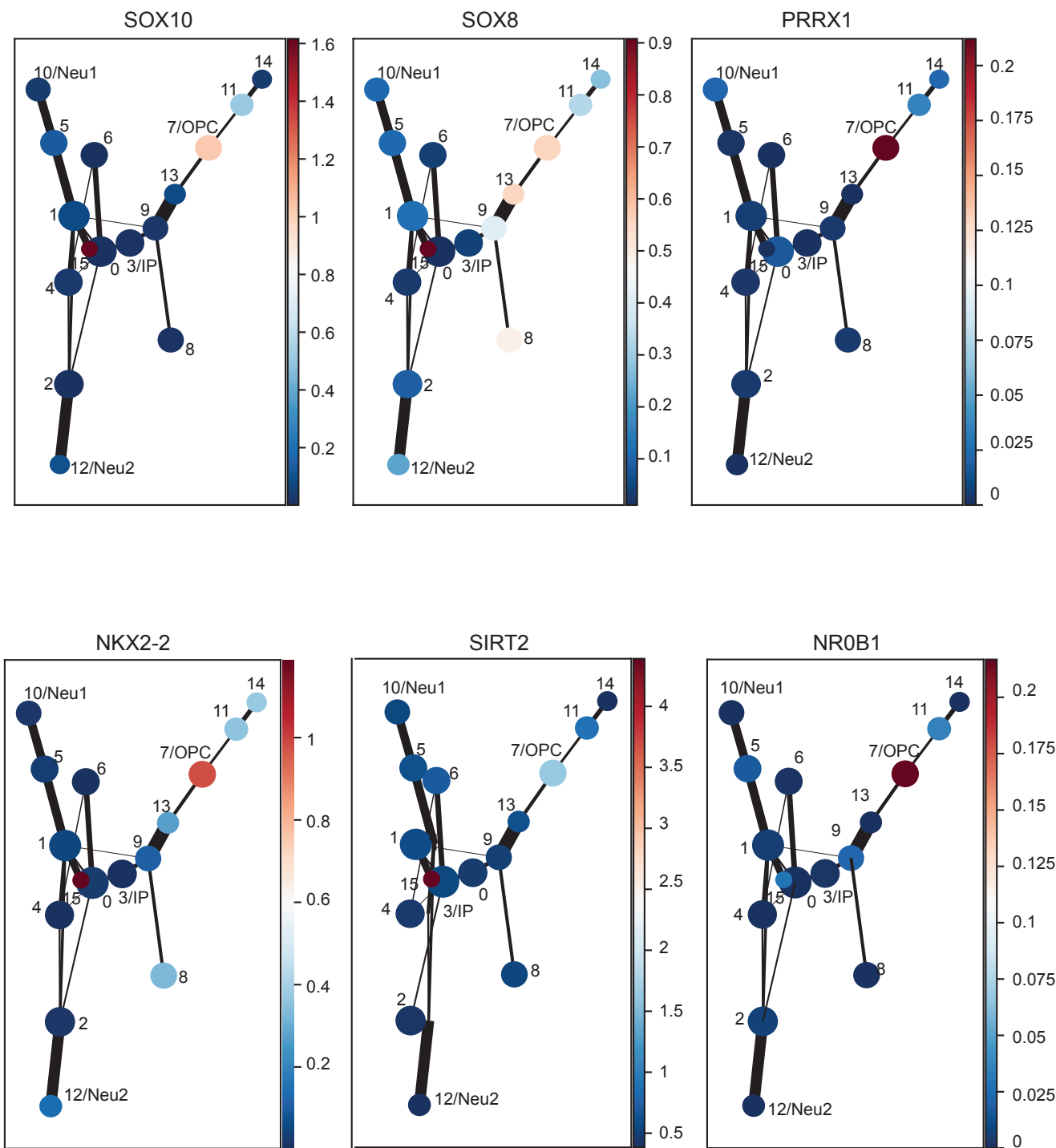

Supplement: Supplementary file 8 [file Image_8.PDF]
